# *In situ* Treatment With Novel Microbiocide Inhibits Methicillin Resistant *Staphylococcus aureus* in a Murine Wound Infection Model

**DOI:** 10.3389/fmicb.2019.03106

**Published:** 2020-01-23

**Authors:** Joseph P. Hoffmann, Jessica K. Friedman, Yihui Wang, James B. McLachlan, Mimi C. Sammarco, Lisa A. Morici, Chad J. Roy

**Affiliations:** ^1^Department of Microbiology and Immunology, Tulane University School of Medicine, New Orleans, LA, United States; ^2^Department of Surgery, Tulane University School of Medicine, New Orleans, LA, United States; ^3^Division of Microbiology, Tulane National Primate Research Center, Covington, LA, United States

**Keywords:** wound infection, wound healing, *Staphylococcus aureus*, luminescent, antimicrobial, microbiocide

## Abstract

Increased prevalence of antibiotic resistance in skin and soft tissue infections is a concerning public health challenge currently facing medical science. A combinatory, broad spectrum biocidal antiseptic has been developed (“ASP”) as a topically applied solution to potential resistant and polymicrobial infected wounds that may be encountered in this context. The ASP-105 designate was evaluated *in vitro* by determining the minimal inhibitory concentration (MIC) and minimal bactericidal concentration (MBC), against different strains of methicillin-resistant *Staphylococcus aureus* (MRSA), resulting estimates of which approximated the positive control (bacitracin). To evaluate *in vivo* microbicide efficacy, we utilized a murine full thickness wound model to study bacterial infection and wound healing kinetics. Mice were experimentally wounded dorsally and infected with bioluminescent MRSA. The infected wound was splinted, dressed and treated topically with either ASP-105, vehicle (-control), or bacitracin. Bacterial burden and wound healing was monitored using an *in vivo* imaging system and evaluation of biofilm formation using scanning electron microscopy of wound dressing. Treatment with ASP-105 significantly reduced bacterial burdens in the first 3 days of infection and inhibited MRSA biofilm formation on the surgical dressing. Notably, treatment with ASP-105 resulted in a sterilizing effect of any detectable MRSA in nearly all (80%; 4/5) of treatment group. All mice receiving vehicle control developed highly MRSA-luminescent and purulent wound beds as a result of experimental infection. The ASP-105 therapy facilitated natural healing in the absence of MRSA infection. Results of this study suggests that that the novel “ASP” combinatory topical antiseptic can be used directly in wounds as a potent, broad-spectrum microbicide against drug resistant *S. aureus* without injury to the wound bed and impediment of natural restorative processes associated with wound healing. Further studies are warranted to test the effectiveness of this biocidal formulation against other recalcitrant bacterial and fungal pathogens in the context of serious wound infections, and to assess utility of use in both clinical and self-treat scenarios.

## Introduction

With the skin being the body’s first line of defense, abrasions and penetrating wounds of the skin typically obtained in combat, a hospital, or even the community are particularly prone to infection. Rates of skin and soft tissue infections (SSTIs) are particularly high as they are among the most common infection in both ambulatory and hospital settings (Hersh et al., 2008; Miller et al., 2015; Kaye et al., 2019). Not only are SSTIs common, but incidence in these infections has continued to rise over the past few decades (Edelsberg et al., 2009). One study found that in the United States each year, there are 3.3 million SSTI diagnoses, which is a 40% increase from the early 2000s (Suaya et al., 2014). Among the bacteria that infect these wounds, the most commonly isolated and clinically relevant is *Staphylococcus aureus* (Lowy, 1998; Tong et al., 2015). *S. aureus* is particularly troubling in a clinical setting as it readily acquires antibiotic resistance mechanisms and has a propensity to form biofilms in the wound bed resulting in chronic infections (Clinton and Carter, 2015). With *S. aureus* infections so prominent in a clinical setting, patients undergoing a surgical procedure are particularly at risk for infection. In the United States surgical site infections are the most common cause of hospital acquired infection (Ban et al., 2017). Currently there are extensive preventative measures taken such as a prophylactic administration of antibiotics and preoperative cleaning of the incision site with an alcohol-containing agent (Anderson et al., 2014). With a rise in antimicrobial resistance and the fact that alcohol alone does not generate lasting protection of the wound alternatives may be needed (Anderson et al., 2014).

Though MRSA was originally restricted to hospital settings, genetically distinct strains emerged in communities in the 1990s leading to new infections with community acquired MRSA (CA-MRSA) (David and Daum, 2010). CA-MRSA infections have now become so common that one study described that from 190 patients with uncomplicated skin and skin structure *S. aureus* infections, 53% were infected with CA-MRSA (Jones et al., 2007). Currently in the United States, the most common empiric antibiotic treatment for *S. aureus* SSTIs is a regimen of systemic treatment with antibiotics such as daptomycin, linezolid, or vancomycin (Stevens et al., 2014; Ramakrishnan et al., 2015). For directed treatment against MRSA, trimethoprim-sulfamethoxazole is typically used (Stevens et al., 2014). Though these drugs are efficacious they can cause adverse systemic side effects and the prevalence of antibiotic resistance limits choices (Ramakrishnan et al., 2015). The topical treatment of wounds post-operatively or in the community with novel antimicrobials are a potential way to both prevent and treat SSTIs.

Novel topical antimicrobials are needed to prevent further spread and development of complications to effectively combat commonly occurring, recalcitrant bacterial infections. Such topical treatments could be administered in a community setting after a cut or abrasion or clinically after surgery to reduce risk of surgical site infection (Reichman and Greenberg, 2009). Topical administration of antimicrobials is advantageous in that it allows for delivery of high concentrations directly to the wound bed, reduces the potential for systemic toxicity, limits the exposure of the drug to normal flora, and permits the use of drugs that may not be administered systemically (Lio and Kaye, 2004; Lipsky and Hoey, 2009).

Currently there is a wide array of wound infection models in mice that can be used to evaluate new topical antimicrobials *in vivo*. These include the tape strip model (Kugelberg et al., 2005; Tatiya-aphiradee et al., 2016), incisional wound model (Lu et al., 2014), burn wound model (Liu et al., 2014; Turner et al., 2014), and a wound model that uses a biopsy punch (Zhao et al., 2010; Thompson et al., 2014; Chhibber et al., 2018). Though all have shown utility in assessing wound bacterial burden, few of the identified models lend themselves to evaluation of candidate topical antimicrobial therapies nor approximate wound healing in humans. Higher phylogeny mammals heal predominantly through re-epithelialization and formation of granulation tissue, while the rodent wound heals mainly through skin contraction (Galiano et al., 2004). This complicates the translatability of wound healing studies using murine species. The full thickness excisional splinted wound model overcomes this shortcoming of rodent models by integration of a wound splinted using sutured silicon torus. The splint prevents healing by contraction, and rather forces re-epithelialization and the formation of granulation tissue, similar to what occurs in humans (Dunn et al., 2013; Wang et al., 2013). The splinted wound model has primarily been used in wound regeneration and repair studies (Du et al., 2017), but in recent years it has been adopted in research to evaluate how the pathophysiology of infection and antimicrobial treatments affects overall natural wound repair and restorative processes (Schierle et al., 2009; Brandenburg et al., 2015).

In this study, the splinted full thickness excisional wound model was used to evaluate the efficacy of a novel topical therapeutic (ASP-105) as a treatment for CA-MRSA wound infection. We demonstrate that treatment with ASP-105 inhibits the growth of CA-MRSA in the wound bed, reduces gross pathology, inhibits biofilm formation, and does not impair the wound healing process.

## Materials and Methods

This work comprises three separate animal studies. Initially, wound healing and bacterial burdens were modeled throughout a 2-week infection by infecting splinted wounds with two strains of bioluminescent *S. aureus* and one strain of bioluminescent *Pseudomonas aeruginosa*. Wound healing and bacterial burdens were monitored by using an *in vivo* imaging system (IVIS) described in this section. Thereafter, the utility of the wound infection model was evaluated by topically treating bioluminescent CA-MRSA infected wounds with bacitracin or vehicle control and quantifying wound healing, bacterial burdens, and gross pathology. The third study investigated the antimicrobial and antibiofilm efficacy of the experimental antimicrobial ASP-105 and its influence on wound healing and gross pathology. The antimicrobial efficacy is additionally tested via the minimum inhibitory concentration (MIC) and minimum bactericidal concentration (MBC) of ASP-105 against clinically relevant strains of *S. aureus in vitro*.

### Bacterial Strains Used for Inoculum Preparation

When developing the wound infection model, three strains of bioluminescent bacteria were used: CA-MRSA strain MW2, *S. aureus* strain Newman, and *P. aeruginosa* strain PAO1. Mice were infected with CA-MRSA in all animal studies that evaluated topical antimicrobials. The bioluminescent Newman and MW2 strains of *S. aureus*, were generated, described, and generously provided by Dr. Roger Plaut at the United States Food and Drug Administration (Plaut et al., 2013). The bioluminescent PAO1 was generated and generously provided by Dr. Michael Schurr at the University of Colorado Denver. CA-MRSA, *S. aureus* strain Newman, and PAO1 were cultured overnight in 5 mL Miller lysogeny broth (LB) (Fisher Scientific) for each discrete wound infection. From the overnight culture, 250 μL was subcultured into 25 mL LB in a 125 mL Erlenmeyer flask, incubated at 37°C and shaken at 233 rpm for 4 h until the culture reached early to mid-exponential growth phase. Cells were collected at an optical density of ≈0.7 at 600 nm [using a SmartSpec^TM^ Plus Spectrophotometer (Biorad, Hercules, CA, United States)]. Cells were washed with sterile 1x phosphate buffered saline (PBS) 2× and resuspended in sterile 1× PBS so that the concentration of bacteria was 1 × 10^6^ colony forming units per mL (CFU/mL), ensuring that 10 μL applied to each wound delivered 1 × 10^4^ CFU dose. Inoculum bacterial dose was verified by serial dilution and standard culture technique using LB agar. For our *in vitro* studies investigating the efficacy of ASP-105 against multiple strains of *S. aureus*, we will utilize the bioluminescent MW2 and Newman strains described above as well as a bioluminescent strain of USA300-0114 developed by Plaut et al. (2013), subspecies Rosenbach ATCC 6538, and a clinical isolate of MRSA obtained at Tulane University School of Medicine.

### Mice Used and Animal Care

All infection studies used female CD1 mice (Charles River Laboratories, Boston, MA, United States). Animals were acquired at 7–10 weeks of age at 24–34 g and provided with food and water *ad libitum*. Animals were maintained on an alfalfa-free diet prior to and throughout the infection period to minimize autofluorescent signal (Inoue et al., 2008). Mice were singly housed post-procedure to prevent cage mates potentially damaging the wound site or accidential autoinoculation of wounds during treatment studies.

## Therapeutic Agents

### Bacitracin

A mixture of related cyclic polypeptides, bacitracin is produced by organisms of the *licheniformis* group of *Bacillus subtilis* var Tracy. Bacitracin has known efficacy against most Gram-positive bacteria, including *S. aureus*. It is synthesized via the activity of non-ribosomal peptide synthetases (NRPSs) and is nearly exclusively used as a topical antibiotic. The bacitracin used in these studies was commercially acquired (Bacitracin Zinc, 500 units/ml, Millipore/Sigma, St. Louis, MO, United States).

### ASP-105

The “ASP” microbicide used in these studies is a broad spectrum biocidal formulated in a phosphate buffer, the componentry of which is detailed elsewhere (Fears et al., 2018). Briefly, the ASP-105 formulation contains a combination of oxychlorine, free ammonium and methylated-ammonium ingredients which possess independent cationic and direct oxidizing activity against a broad range of microorganisms in a non-competitive manner at physiological pH (pH = 7.14).

### *In vitro* MIC and MBC Analysis of Therapeutic Agents

Cultures of each strain of *S. aureus* were grown overnight in Mueller-Hinton Broth then diluted 1:100. A volume of 40 μL was added to the wells of a 96 well plate from this dilution. Next, 10 μL of ASP-105, ASP-106 (a control which consists of all the components of ASP-105 without any of the active ingredients), and Bacitracin were all added in triplicate and serially diluted by a factor of five to test for inhibitory and bactericidal activity. Plates were incubated overnight at 37°C and read at 600 nm on a plate reader to determine inhibitory activity. Once each plate was read, 3 μL from each well was spot plated on LB agar and incubated overnight at 37°C to determine the minimum bactericidal concentration. The MIC and MBC for each strain and antimicrobial was repeated three times. The MIC and MBC is reported as the dilution factor as ASP-105 is composed of multiple active ingredients.

### Full Thickness Splinted Punch Wound Model

All experimental procedures performed on animals were previously approved by the Tulane University Institutional Animal Care and Use Committee (P0131). On day 0, mice were wounded and the wound bed directly infected with bacteria. Animals were anesthetized by an intraperitoneal (IP) injection of ketamine (90 mg/kg) and xylazine (10 mg/kg) mixture. A separate IP injection of buprenorphine (0.05 mg/kg) was also administered to prophylactically accommodate for pain. Once fully anesthetized, the dorsum was shaved and the exposed skin was scrubbed with chlorhexidine to sanitize the surface. Thereafter, a full-thickness wound was generated using a 5 mm biopsy punch (Integra Miltex) by application of pressure on the dorsum below the base of the skull and between the solar plexus, generating a 5 mm circular wound outline. The light perforation was then excised and the skin was cut through the epidermis, dermis, and panniculus carnosis exposing the muscle beneath. Thereafter, a 10 mm silicone torus coated in surgical adhesive was placed over the wound. Tegaderm (3 M Healthcare) was placed over the silicone torus covering the exposed wound bed and the torus was secured in place using 4-0 braided silk interrupted sutures (Ethicon) fortified with additional surgical adhesive. An insulin syringe (BD Biosciences) was used to experimentally infect the wound by penetrating the tegaderm and expressing directly onto the wound bed. Mice were monitored daily to access bacterial burdens, wound closure, and weight change for the duration of the study until sacrifice on day 14. Daily injections of buprenorphine (0.05 mg/kg) were performed every 12 h for the first 2 days post-innoculation with repeated doses given later if needed to accommodate for any postsurgical pain.

### Treatment

Animals were allowed to recover for 4 h post wounding surgery and inoculation and then anesthetized via inhalation (2.5%, isoflurane, VetOne, Boise, ID, United States). Thereafter either ASP-105 (undilute), Bacitracin (500 U/mL), or vehicle control was administered via 20 μL injection through the tegaderm covering to separate groups (*n* = 5). For the second animal study that exclusively use bacitracin topically, the vehicle control is 1×PBS. For the third animal study that investigates the efficacy of ASP-105, the compound ASP-106 is used as a vehicle control. Treatments were provided every 8 h for the first 3 days of infection (total of nine treatments).

### Observations and Clinical Scoring

Animals were monitored and weighed daily to day +14 to assess health in response to infection and treatment. Individual wounds were examined daily, clinical status assessed, and categorically scored using the following criteria: 0 = no redness, swelling, or discharge; 1 = light inflammation and some discharge; 2 = redness, swelling, discharge, and/or discoloration; 3 = heavy inflammation, discoloration, and purulence.

### Quantification of Bacterial Burdens and Wound Closure

To determine bacterial burdens within the wound over time and examine the rate at which the wound was healing, mice were imaged daily using an *in vivo* imaging system (IVIS)-XMRS (PerkinElmer). The IVIS is capable of detecting and quantifying bioluminescent signals used to track the infection progression of the bioluminescent bacteria within the wound. During use, mice were anesthetized via inhalation of 2.5% isoflurane and imaged individually for 60 s. Resulting images were analyzed using the IVIS Lumina Living Image Software (PerkinElmer). A circular region of interest (RoI) was electronically captured over each wound bed to quantify bioluminescence and bacterial burdens, which was used for every mouse every day. Relative luminescence within the RoI in units of radiance (photons/centemeter^2^/steradian/second) is interpolated by companion system software. Animals were humanely euthanized on day 14 via CO_2_ asphyxiation, and tissues sampled to further quantify bacterial burdens by excising the wound, which extended roughly 5 mm beyond the wound edge. Tissue samples were mechanically disrupted in 1 mL sterile 1x PBS, serially diluted, and plated on LB agar. Resulting bacterial colonies were imaged (IVIS) to verify the presence of CA-MRSA. In addition, the spleen and liver were removed and processed in a similar manner for determination of whether the bacteria had disseminated from the wound.

The rate and progression of wound healing was monitored for animals using custom RoIs traced along the epithelial lip of each wound (IVIS) that generated a wound area estimate. Wound area measurements were performed daily beginning upon the removal of the tegaderm covering, and provided a quantitative assessment of the rate and progression of wound healing over time.

### Scanning Electron Microscopy (SEM) of MRSA Biofilm

The tegaderm wound dressing was removed from each animal +3 days post-infection and imaged using SEM. Briefly, a sample of the tegaderm was adhered to a hydroxyapatite disk (5 mm), and fixed in glutaraldehyde (2.5%, Electron Microscopy Sciences) overnight at 4°C. Thereafter, the disk was washed 3x in distilled H_2_O, and then dehydrated in 5 min sequential washes of ETOH (25, 50, 75, 90, and 100%). Samples were then critical point dried (autosamdri^®^-814, Tousimis, Rockville, MD, United States), coated in carbon, and imaged using SEM (Hitachi S-4800 FEG CRYO-SEM). Representative images of each sample (*n* = 10) at both 2 Kx and 10 Kx were taken at an operational voltage of 3 Kv.

### Statistical Analysis

All statistical analysis was performed in Graphpad Prism version 6 (Graphpad Software, San Diego, CA, United States). For all data sets, we performed a *t*-test at each time point after applying the Bonferroni correction for multiple comparisons. For analysis of average radiance data we exported the average radiance values generated by Living Image Software into Graphpad Prism version 6. We then performed a log transformation and conducted a *t*-test on the transformed values as described above.

## Results

### Wound Infection Model Development

**FIGURE 1:**
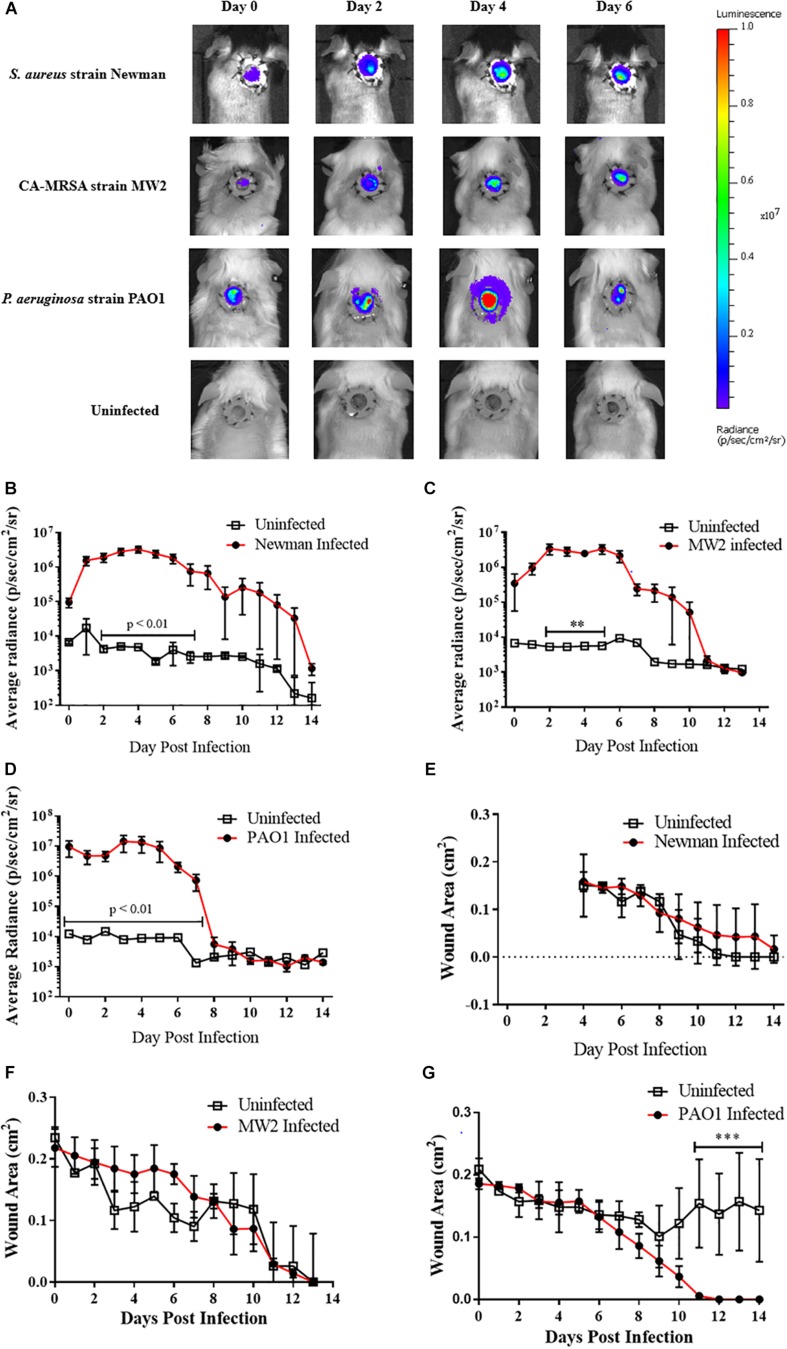
Murine full thickness splinted wound model to study infection progression and wound healing: **(A)** Representative images during first week of infection in mice infected with 10^5^ CFU bioluminescent strains of either *Staphylococcus aureus* [Newman (*N* = 5); MW2 (*N* = 3)] or *Pseudomonas aeruginosa* [PAO1 (*N* = 4)]. For each infection study 1–2 mice were wounded but inoculated with PBS as a negative control; peak luminescence observed at +4 days for most infections. **(B–D)** Radiance plots (mean ±SEM) of mouse wound bed over 14 days infected with either *S. aureus* strain Newman **(B)**, CA-MRSA strain MW2 **(C)**, or *P. aeruginosa* **(D)**. **(E–G)** Wound area measurements (mean ±SEM) during 14 days infection for mice infected with **(E)** *S. aureus* strain Newman, **(F)** CA-MRSA strain MW2, and **(G)** *P. aeruginosa.* For each infection, PBS was used as vehicle control in wounds in additional animals. Statistical difference tested by Student’s *T*-test [(^∗∗^*p* < 0.01, ^∗∗∗^*p* < 0.001). For **(B)** days 2–4, 6, *p* < 0.001; day 5, *p* < 0.0001; and day 7, *p* < 0.01. For **(D)** days 0, 2, *p* < 0.001; days 1, 3–4, *p* < 0.0001; and days 5–7, *p* < 0.01.

Two bioluminescent strains of *S. aureus* Newman and CA-MRSA MW2, and a luminescent strain of *P. aeruginosa* (PAO1) were used as exemplars to assess the feasibility of experimental infection of wounds and whether there was enough fidelity in the model to provide semi-quantitative representation of bacterial contamination. Measurable luminescence was detectable immediately post-inoculation for all bacterial species and individual strains attempted with clear homogenous spread within the wound bed by +2 days (Figure 1A). The *P. aeruginosa* infection emitted a much stronger, definable signal than either *S. aureus* strains tested, suggesting that this species proliferated in the wound bed successfully, with this specific strain potentially producing more luciferase which registered a brighter radiance. All bacteria followed a similar infectious time course, proliferating rapidly within the first 2 days post-inoculation (Figures 1B–D), and then peaking +3–4 days, with a plateau and natural resolution and clearing initiating by +7 days. All experimentally infected mice had cleared the infection to be below the limit of detection and matched the values for the uninfected sentinels by +14 days (Figures 1B–D). Verification of natural clearance of infection was performed by excising, homogenizing and plating tissue from wound beds, which resulted in no bioluminescent bacteria recovery from the homogenized tissue (data not shown).

The rate of wound closure was not statistically different when comparing infected with uninfected mice for any bacterial species or strain (Figures 1E–G). CA-MRSA-infected animals trended toward a slower wound closure when compared with their uninfected counterparts in the first week of infection (Figure 1F), with corresponding bacterial burdens decreasing by +7 days post-infection. Opportunistic infection of an experimentally wounded, but uninfected animal performing self-grooming activities delayed wound healing when compared with infected mice (Figure 1G). Weight was also monitored daily as an endpoint of wound status and healing, and although there was no correlation with wound closure (Supplementary Figure S1), slight weight reduction was recorded immediately post-procedure. Collectively, the infected wound mouse model as described was demonstratable to a self-limited, highly localized proliferative bacterial infection appropriate for use in topical antimicrobial evaluation studies.

### Infected Wound Model for Evaluation of Antimicrobial Agents

**FIGURE 2:**
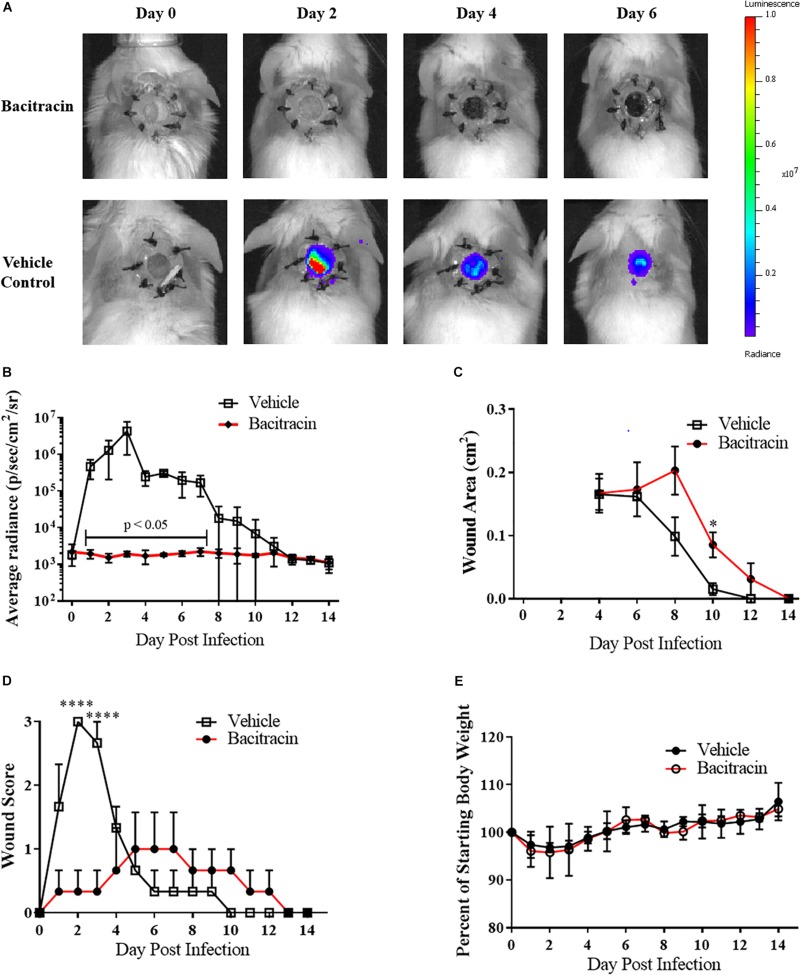
Topical antimicrobials can be tested using the splinted wound model: **(A)** Images of mice infected with 10^4^ CFU CA-MRSA and treated with either 500 U/mL bacitracin or vehicle control (*N* = 3 for each group) over 7 days where treatment eliminated detectable bioluminescence. **(B)** Radiance plots (mean ±SEM) of mouse wound bed. **(C)** Wound area measurements (mean ±SEM). **(D)** Gross wound pathology categorical scoring (mean ±SEM). **(E)** Daily mean group weights. Statistical difference by Student’s *T*-test (^∗^*p* < 0.05, ^∗∗∗∗^*p* < 0.0001). For **(B)** days 1, 3–4, 7, *p* < 0.001; day 2, *p* < 0.05; day 5, *p* < 0.0001; day 6, *p* < 0.01.

To evaluate the utility of the infected wound model for the assessment of topical antimicrobials, we next topically treated CA-MRSA infected mice with either the FDA-approved non-prescriptive (OTC) antibiotic bacitracin or a vehicle control. No bacitracin-treated mice had detectable burdens for the entirety of post infection observation period (+14 days), while all (100%, 8/8) control-treated mice resulted in detectable CA-MRSA, with bacteria covering the entirety of the wound bed by the second day of infection, evidenced by the IVIS imaging (Figure 2A), with infection kinetics similar to modeling efforts (Figure 2B). Interestingly, the bacitracin-treated animals tended to lag in rapidity of contraction, with significant differences observed on day 10 (Figure 2C). Clinical examination of the wounds yielded stark differences between groups. Bacitracin-treated animals showed minimal signs of infection, with no purulence, erythema, or other inflammation where in contrast controls developed purulent, inflamed wound beds by +2 days of infection (Figure 2D). Treatment and infection status had no effect on weight change (Figure 2E).

### The Novel Microbicide ASP-105 Is Bactericidal to Clinically Relevant MRSA Strains *in vitro* and *in vivo*

The MIC and MBC of ASP-105 was initially determined using a panel of *S. aureus* strains (Table 1), including laboratory *S. aureus* ATCC 6538, bioluminescent *S. aureus* strain Newman, bioluminescent CA-MRSA strains MW2 and USA300-0114, and a MRSA clinical isolate obtained from the Tulane University Hospital. The ASP-105 formulation demonstrated potent *in vitro* inhibitory activity against all five strains of *S. aureus* tested, and inhibited both CA-MRSA strains (1:125 dilution) and the Tulane Hospital clinical isolate (1:25 dilution). The resulting MICs for bacitracin against all but USA300-0114 tested (67 μg/mL) approximated the dilution (and subsequent dose) used in the murine wound treatment experiments, which equated to the inhibitory capacity of bacitracin against MRSA *in vivo*. Similarly, ASP-105 was bactericidal at a 1:25 dilution in MBC determination for four of the five strains tested, which roughly equated to the dilution factor for bacitracin against CA-MRSA. Interestingly, our USA300-0114 strain was resistant to bacitracin when compared with the other *S. aureus* strains. With bacitracin against USA300-0114, we observed a much higher inhibitory concentration of 333 μg/mL and no bactericidal activity. In contrast, ASP-105 possessed the same activity against USA300-0114 as it did the other strains *S. aureus*.

**TABLE 1:** ASP-105 is both inhibitory and bactericidal to multiple strains of *S. aureus in vitro.*

Minimum inhibitory concentration			

*S. aureus* strain	ASP-105	Control	Bacitracin (μg/mL)
*S. aureus* ATCC 6538 subspecies Rosenbach	1:125	>1:5	67
MRSA (Tulane clinic isolate)	1:25	>1:5	67
Bioluminescent CA-MRSA strain MW2	1:125	>1:5	67
Bioluminescent CA-MRSA strain USA300-0114	1:125	>1:5	333
Bioluminescent *S. aureus* strain Newman	1:125	>1:5	67

**Minimum bactericidal concentration**			

***S. aureus* strain**	**ASP-105**	**Control**	**Bacitracin (μg/mL)**

*S. aureus* ATCC 6538 subspecies Rosenbach	1:25	>1:5	67
MRSA (Tulane clinic isolate)	1:25	>1:5	67
Bioluminescent CA-MRSA strain MW2	1:25	>1:5	333
Bioluminescent CA-MRSA strain USA300-0114	1:25	>1:5	>8,330
Bioluminescent *S. aureus* strain Newman	1:5	>1:5	333

*MIC and MBC for ASP-105 and the control are reported in dilution factor.*

**FIGURE 3:**
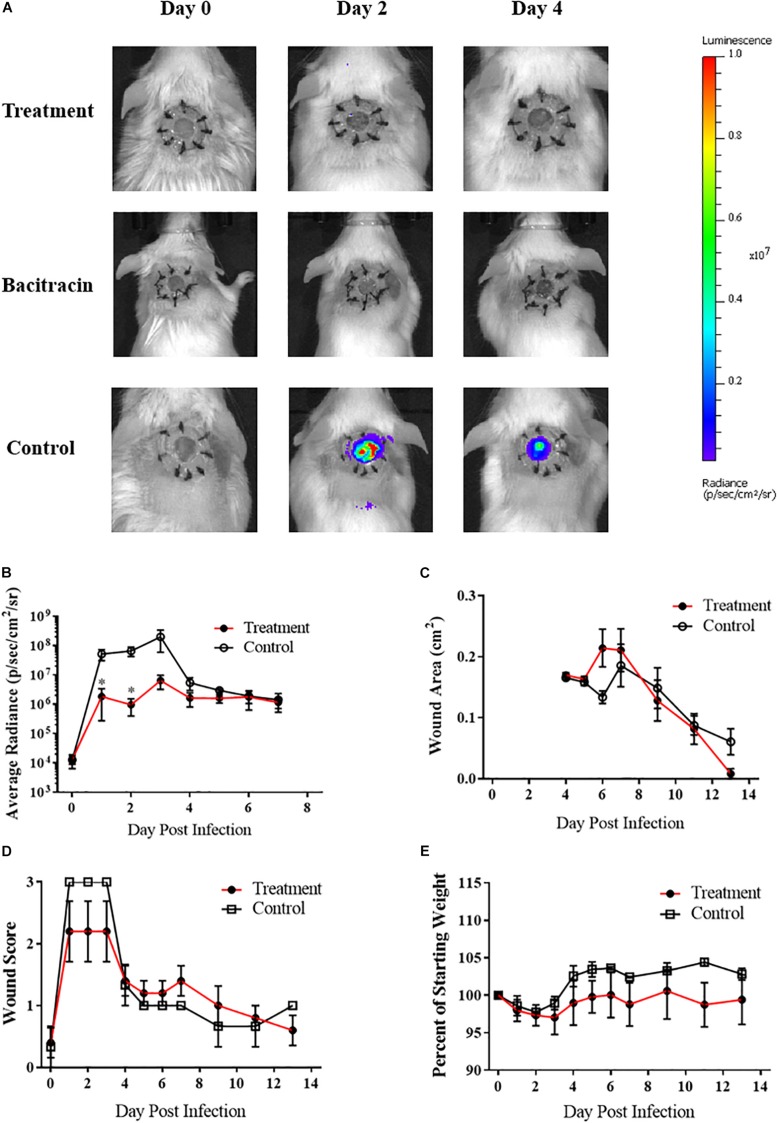
ASP-105 Biocide Inhibits the growth of CA-MRSA in wounds: **(A)** Images of mice infected with 10^4^ CA- MRSA treated with either ASP-105, Bacitracin, or vehicle through +3 days (*N* = 5 for each group). Neither bacitracin nor ASP-105-treated animals show any bioluminescence. **(B)** Radiance plots (mean ±SEM) of mouse wound bed over 7 days infected with CA-MRSA ASP-105-treated animals show significantly lower burdens through treatment. **(C)** Wound area measurement (mean ±SEM) when treated with either ASP-105 or vehicle initiating after surgical dressing removal, ASP-105 treatment had no significant impact on wound closure. **(D)** Gross wound categorical scoring (mean ±SEM). **(E)** No significant difference observed in daily mean group weights. Statistical difference by Student’s *T*-test (^∗^*p* < 0.05).

After verifying ASP-105’s antimicrobial activity against CA-MRSA *in vitro*, we tested the compound *in vivo* using our established wound model. Clear inhibition of CA-MRSA was observed in the wound bed over the 3 days of treatment in ASP-105-treated mice with burdens not appearing on a heatmap when applied to the same scale as vehicle control treated mice (Figure 3A). Nearly all (4/5; 80%) of ASP-105-treated mice had a readily distinguishable reduction of bacterial burdens through the duration of the 3-day treatment period resulting in significantly lower burdens on the first 2 days of treatment (Figure 3B). Some of the treated mice (3/5; 60%), however, showed slight increases in bacterial luminescence upon the cessation of treatment. Vehicle control-treated animals demonstrated unfettered bacterial growth similar to the pattern of what was observed when establishing the CA-MRSA infection wound model. The gross clinical pathology categorical scoring showed vehicle control-treated mice experiencing purulent, clearly infected wounds +2 to +3 days post infection procedure. In contrast, 3 of five (3/5; 60%) ASP-105-treated animals developed gross clinical changes associated with infection (Figure 3D). Wound closure and weight change were not significantly different among experimental groups (Figures 3C,E), suggesting that treatment with ASP-105 does not deter wound closure or negatively impact the mice.

**FIGURE 4:**
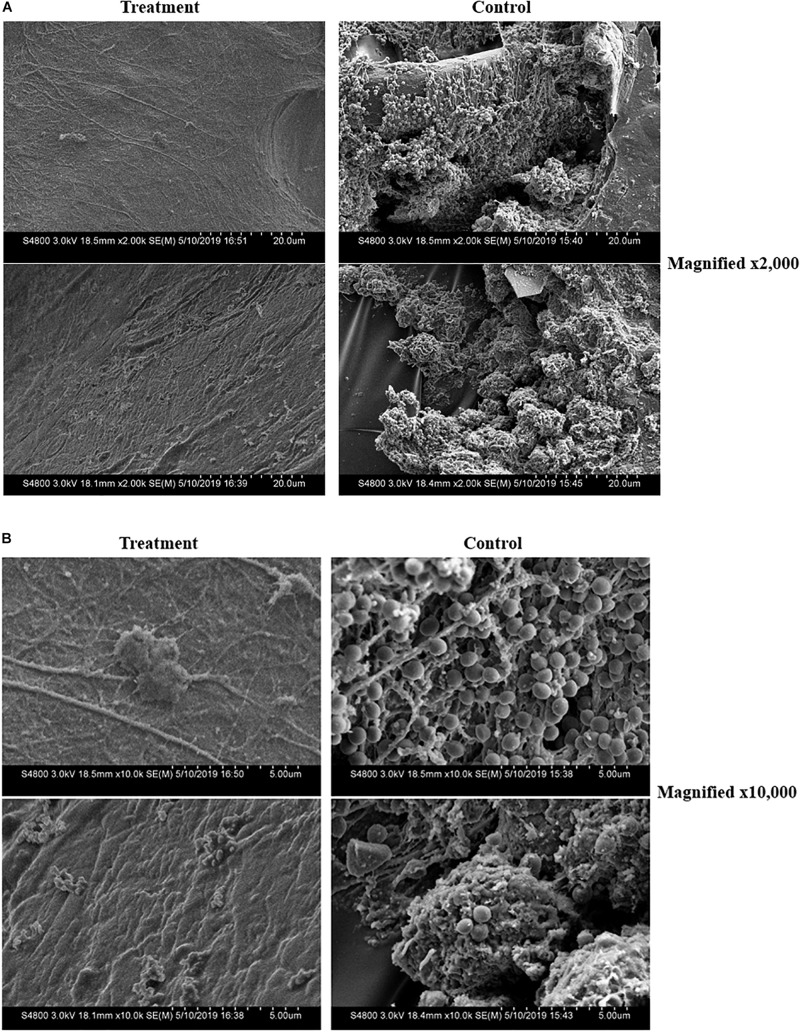
ASP-105 prevents the formation of MRSA biofilms on surgical wound dressing. SEM images of removed Tegaderm +3 days post infection in ASP-105-treated or vehicle controls (*N* = 5 per group). Images shown at 2,000x **(A)** and 10,000x **(B)** magnification. Dressing from treatment animals show no identifiable bacteria contrasting with control animals which resulted in dense biofilm with clearly definable staphylococci **(B)**.

Analysis of the Tegaderm overlay via SEM illustrated a clear inhibition of biofilm formation on the surgical covering placed over the mouse wounds in animals treated with either ASP-105 or bacitracin. In contrast, the covering on vehicle-treated controls showed a biofilm network composed of densely clustered staphylococcal bacteria nestled within extensive extracellular polysaccharide matrix (Figures 4A,B). Tegaderm surgical coverings from either ASP-105 or bacitracin-treated mice were devoid of biofilm, and mostly cellular debris were the only identifiable components in the fields examined (Figures 4A,B).

## Discussion

Skin and soft tissue infections are incredibly common, with one study citing that they account for up to 14 million outpatient visits to hospitals in the United States annually (Hersh et al., 2008). Given that many skin and soft tissue infections are self-limiting and do not result in hospital visits, their prevalence is likely much higher. *Staphylococcus aureus* is the most common cause of skin and soft tissue infections, and isolates with antimicrobial resistance have become more prevalent in community settings (Turner et al., 2019). One study found that in the United States, MRSA could have a colonization rate of up to 3% (Currie et al., 2008; Shaw et al., 2013). With increasing colonization rate of MRSA there is a greater risk of developing drug resistant wound infections. Thus, it is imperative to not only develop novel antimicrobial therapies, but also utilize the most appropriate models to investigate wound infections and healing. With our study, we have modeled infection progression and wound healing in full thickness wounds infected with bioluminescent antibiotic resistant bacteria. Further, we have employed this model to evaluate the efficacy of the novel microbicide ASP-105.

Given the deficit in novel treatments to antibiotic resistant bacterial infection, the *in vivo* efficacy of ASP-105 against CA-MRSA in a full thickness wound infection is our most important finding. This was further supplemented by our *in vitro* results in Table 1, where we see ASP-105 possesses potent activity against four different clinically relevant strains of *S. aureus* in addition to a clinical isolate with some variability from strain to strain. Though it is essential to verify with *in vivo* experiments, our *in vivo* data coupled with the *in vitro* results in Table 1, suggests that ASP-105 may possess *in vivo* efficacy against other strains of *S. aureus*. We found that topical application of ASP-105 inhibited CA-MRSA growth in the wound bed throughout the duration of the 3-day treatment period (3A). As we demonstrated in 1C, mouse wounds infected with CA-MRSA achieve their peak bacterial load within the first 2 to 3 days of infection. By significantly inhibiting initial burdens, our microbicide limited disease severity. Further adding to the significance in ASP-105’s antimicrobial efficacy is its ability to inhibit the formation of biofilm on the Tegaderm wound dressing. This efficacy against biofilms has very clear clinical applications. Surgical implants and post-surgical dressings are particularly susceptible to biofilm growth (Arciola et al., 2012; Bhattacharya et al., 2015). Once a biofilm has been established, it can be difficult to eradicate. It has been shown that bacteria in a biofilm can withstand antibiotics at up to 1,000× the minimum inhibitory concentration (Ceri et al., 1999). With this in mind, ASP-105 could have successful use as a post-operative antiseptic.

Along with validating a promising new microbicide we have also established a wound infection model for tracking both infection progression and wound healing in individual mice over time. This was done by utilizing bioluminescent bacteria in conjunction with a silicon ring wound splint. The use of bioluminescent bacteria was optimal as it allowed for the day to day tracking of bacterial burdens in individual mice. Further this decreased the number of mice needed for infection studies as it eliminated the need to sacrifice mice at specific time points for bacterial burdens. The use of bioluminescent bacteria to track wound infections has been growing in popularity and become a more common practice (Guo et al., 2013; Fila et al., 2016; Agostinho Hunt et al., 2017). Many wound infection studies, however, lack the use of silicon splints. These splints prevent mice from healing by contracting their skin. Due to the splinting, the wound healing becomes dependent on re-epithelization and the development of granulation tissue, which more accurately mimics the healing process in humans (Dunn et al., 2013). Because of this, the splinted full thickness wound model is the most ideal and clinically relevant rodent model for the study of wound healing kinetics. The tracking of bioluminescent *P. aeruginosa* in conjunction with wound healing in a splinted wound model has been conducted previously by another group (Agostinho Hunt et al., 2017; Hunt et al., 2017). Our study tracking *P. aeruginosa* matches their observations by seeing peak bacterial burdens on day 3–4 of infection and healing over the course of a 2-week period. To our knowledge, this is the first-time wound infection with bioluminescent *S. aureus*, antibiotic resistant or otherwise, has been tracked while also utilizing the splinted wound model. Establishing a model to study infection and healing kinetics is especially important for *S. aureus*, as it is the leading cause of skin and soft tissue infections (Turner et al., 2019). Additionally, with both bacitracin and ASP-105 we have demonstrated that this infection model can be utilized to evaluate topical antimicrobial treatments and their influence on bacterial burdens, wound healing, and overall health. Both treatments generated readily observed decreases in luminescence with little effect on healing. Bacitracin treated mice tended to have a decreased rate of wound healing, but his could be due to mild toxicity from repeated doses of the antibiotic.

Moving forward, there is still much that can be studied with both our novel microbicide ASP-105 and the wound infection model itself. One key limitation of our study was the use of only female mice. Though we see that ASP-105 causes clear inhibition of CA-MRSA infections, follow-up studies using both male and female mice will strengthen rigor of our initial findings. The inclusion of both gender is particularly important in the context of wound healing as it has been shown there is a disparity of growth factor expression between sexes which may lead to different wound healing outcomes (Castleman et al., 2017; Vermillion et al., 2018). Now that we have demonstrated ASP-105’s efficacy *in vivo*, future studies can be conducted to determine the most appropriate frequency and concentration of dose applied. A higher dose that requires less frequent application is ideal from a clinical perspective, but it will also serve to reduce the number of times animals undergo anesthesia for treatments in preclinical studies. To further highlight our respect and concern for animal welfare in these studies we can also use the data we have generated to reduce the number of times animals are imaged, as from our modeling data it appears that we can decrease frequency of imaging after the clearance of infection. Additionally, data generated in this study can be used in power calculations for future studies to reduce the number of animals used.

We have demonstrated ASP-105’s efficacy against CA-MRSA, but further tests need to be conducted using other common causative agents of skin and soft tissue infections such as *P. aeruginosa*, *Acinetobacter baumannii*, and *Streptococcus pyogenes* to determine if our microbicide possesses broad spectrum activity *in vivo*. Wound healing kinetic data demonstrated that ASP-105 did not inhibit wound closure nor induced any gross changes indicative of an inflammatory or adverse effect when applied directly to wound bed. Though there was a slight decrease in weight in the first few days post infection, this was likely due to the wound and infection model itself as all the infection groups in Figure 1, have similar patterns of weight loss. When examining the infection model itself, there are many potential applications in studying the interplay between bacterial burdens and the wound healing process itself. This splinted wound infection model is ideal for studying how infection status influences they dynamics of inflammation, healing, and tissue organization in the wound healing process.

## Data Availability Statement

The raw data supporting the conclusions of this article will be made available by the authors, without undue reservation, to any qualified researcher.

## Ethics Statement

The animal study was reviewed and approved by the Tulane University, Institutional Animal Care and Use Committee.

## Author Contributions

JH, JF, and YW performed the experiments described in this manuscript. JH conducted all the data analysis with feedback from co-authors. MS and LM conceived and facilitated the development of the wound infection model. JM provided essential reagents to the experiments. JH, CR, and LM wrote the manuscript.

## Conflict of Interest

CR is part owner and holds a financial interest in Asepticys LLC, the supplier of the ASP reagents that were used in this study. The remaining authors declare that the research was conducted in the absence of any commercial or financial relationships that could be construed as a potential conflict of interest.

## Supplementary Material

FIGURE S1 Wound infection with CA-MRSA and *Pseudomonas aeruginosa* does not affect weight change. Mean weights of mice infected with 10^5^ CFU of either CA-MRSA (*N* = 3) **(A)** or *P. aeruginosa* (*N* = 4) **(B)**.

## References

[B1] Agostinho Hunt A. M. Gibson J. A. Larrivee C. L. O’Reilly S. Navitskaya S. Needle D. B. (2017). A bioluminescent *Pseudomonas aeruginosa* wound model reveals increased mortality of type 1 diabetic mice to biofilm infection. *J. Wound Care* 26 S24–S33. 10.12968/jowc.2017.26.Sup7.S24 28704171

[B2] Anderson D. J. Podgorny K. Berríos-Torres S. I. Bratzler D. W. Dellinger E. P. Greene L. (2014). Strategies to prevent surgical site infections in acute care hospitals: 2014 update. *Infect. Control Hosp. Epidemiol.* 35 605–627. 10.1086/676022 24799638 PMC4267723

[B3] Arciola C. R. Campoccia D. Speziale P. Montanaro L. Costerton J. W. (2012). Biofilm formation in *Staphylococcus* implant infections. a review of molecular mechanisms and implications for biofilm-resistant materials. *Biomaterials* 33 5967–5982. 10.1016/j.biomaterials.2012.05.031 22695065

[B4] Ban K. A. Minei J. P. Laronga C. Harbrecht B. G. Jensen E. H. Fry D. E. (2017). American college of surgeons and surgical infection society: surgical site infection guidelines, 2016 update. *J. Am. Coll. Surg.* 224 59–74. 10.1016/j.jamcollsurg.2016.10.029 27915053

[B5] Bhattacharya M. Wozniak D. J. Stoodley P. Hall-Stoodley L. (2015). Prevention and treatment of *Staphylococcus aureus* biofilms. *Expert Rev. Anti. Infect. Ther.* 13 1499–1516. 10.1586/14787210.2015.1100533 26646248 PMC5142822

[B6] Brandenburg K. S. Calderon D. F. Kierski P. R. Brown A. L. Shah N. M. Abbott N. L. (2015). Inhibition of *Pseudomonas aeruginosa* biofilm formation on wound dressings. *Wound Repair. Regen.* 23 842–854. 10.1111/wrr.12365 26342168 PMC4980578

[B7] Castleman M. J. Pokhrel S. Triplett K. D. Kusewitt D. F. Elmore B. O. Joyner J. A. (2017). Innate sex bias of *Staphylococcus aureus* skin infection is driven by α-hemolysin. *J. Immunol.* 200 657–668. 10.4049/jimmunol.1700810 29222165 PMC5760295

[B8] Ceri H. Olson M. E. Stremick C. Read R. R. Morck D. Buret A. (1999). The calgary biofilm device: new technology for rapid determination of antibiotic susceptibilities of bacterial biofilms. *J. Clin. Microbiol.* 37 1771–1776. 10325322 10.1128/jcm.37.6.1771-1776.1999 PMC84946

[B9] Chhibber S. Kaur J. Kaur S. (2018). Liposome entrapment of bacteriophages improves wound healing in a diabetic mouse MRSA infection. *Front. Microbiol.* 9:561 10.3389/fmicb.2018.00561 PMC5884882 29651276

[B10] Clinton A. Carter T. (2015). Chronic wound biofilms: pathogenesis and potential therapies. *Lab. Med.* 46 277–284. 10.1309/LMBNSWKUI4JPN7SO 26489671

[B11] Currie A. Davis L. Odrobina E. Waldman S. White D. Tomassi J. (2008). Sensitivities of nasal and rectal swabs for detection of methicillin-resistant *Staphylococcus aureus* colonization in an active surveillance program. *J. Clin. Microbiol.* 46 3101–3103. 10.1128/JCM.00848-848 18614650 PMC2546770

[B12] David M. Z. Daum R. S. (2010). Community-associated methicillin-resistant *Staphylococcus aureus*: epidemiology and clinical consequences of an emerging epidemic. *Clin. Microbiol. Rev.* 23 616–687. 10.1128/CMR.00081-89 20610826 PMC2901661

[B13] Du P. Suhaeri M. Ha S. S. Oh S. J. Kim S. H. Park K. (2017). Human lung fibroblast-derived matrix facilitates vascular morphogenesis in 3D environment and enhances skin wound healing. *Acta Biomater.* 54 333–344. 10.1016/j.actbio.2017.03.035 28351680

[B14] Dunn L. Prosser H. C. G. Tan J. T. M. Vanags L. Z. Ng M. K. C. Bursill C. A. (2013). Murine model of wound healing. *J. Vis. Exp.* 75 1–6. 10.3791/50265 23748713 PMC3724564

[B15] Edelsberg J. Taneja C. Zervos M. Haque N. Moore C. Reyes K. (2009). Trends in US hospital admissions for skin and soft tissue infections. *Emerg. Infect. Dis.* 15 1516–1518. 10.3201/eid1509.081228 19788830 PMC2819854

[B16] Fears A. C. Metzinger R. C. Killeen S. Z. Reimers R. S. Roy C. J. (2018). Comparative in vitro effectiveness of a novel contact lens multipurpose solution on *Acanthamoeba castellanii*. *J. Ophthalmic Inflamm. Infect.* 8:19. 10.1186/s12348-018-0161-8 30357549 PMC6200833

[B17] Fila G. Kasimova K. Arenas Y. Nakonieczna J. Grinholc M. Bielawski K. P. (2016). Murine model imitating chronic wound infections for evaluation of antimicrobial photodynamic therapy efficacy. *Front. Microbiol.* 7:1258. 10.3389/fmicb.2016.01258 27555843 PMC4977341

[B18] Galiano R. D. Michaels V. J. Dobryansky M. Levine J. P. Gurtner G. C. (2004). Quantitative and reproducible murine model of excisional wound healing. *Wound Repair. Regen.* 12 485–492. 10.1111/j.1067-1927.2004.12404.x 15260814

[B19] Guo Y. Ramos R. I. Cho J. S. Donegan N. P. Cheung A. L. Miller L. S. (2013). *In vivo* bioluminescence imaging to evaluate systemic and topical antibiotics against community-acquired methicillin-resistant *Staphylococcus aureus*-infected skin wounds in mice. *Antimicrob. Agents Chemother.* 57 855–863. 10.1128/AAC.01003-12 23208713 PMC3553733

[B20] Hersh A. L. Chambers H. F. Maselli J. H. Gonzales R. (2008). National trends in ambulatory visits and antibiotic prescribing for skin and soft-tissue infections. *Arch. Intern. Med.* 168 1585–1591. 10.1001/archinte.168.14.1585 18663172

[B21] Hunt A. M. A. Gibson J. A. Larrivee C. L. O’Reilly S. Navitskaya S. Busik J. V. (2017). Come to the light side: in vivo Monitoring of *Pseudomonas aeruginosa* biofilm infections in chronic wounds in a diabetic hairless murine model. *J. Vis. Exp.* 128:e55991. 10.3791/55991 29053700 PMC5752399

[B22] Inoue Y. Izawa K. Kiryu S. Tojo A. Ohtomo K. (2008). Diet and abdominal autofluorescence detected by in vivo fluorescence imaging of living mice. *Mol. Imaging* 7 21–27. 10.2310/7290.2008.0003 18384720

[B23] Jones R. N. Nilius A. M. Akinlade B. K. Deshpande L. M. Notario G. F. (2007). Molecular characterization of Staphylococcus aureus isolates from a 2005 clinical trial of uncomplicated skin and skin structure infections. *Antimicrob. Agents Chemother.* 51 3381–3384. 10.1128/AAC.01588-1586 17576829 PMC2043172

[B24] Kaye K. S. Petty L. A. Shorr A. F. Zilberberg M. D. (2019). Current epidemiology, etiology, and burden of acute skin infections in the United States. *Clin. Infect. Dis.* 68 S193–S199. 10.1093/cid/ciz002 30957165 PMC6452002

[B25] Kugelberg E. Norström T. Thomas K. Duvold T. Andersson D. I. Norstro T. (2005). Establishment of a superficial skin infection model in mice by using *Staphylococcus aureus* and *Streptococcus pyogenes* establishment of a superficial skin infection model in mice by using *Staphylococcus aureus* and *Streptococcus pyogenes*. *Antimicrob. Agents Chemother.* 49 3435–3441. 10.1128/AAC.49.8.3435 16048958 PMC1196267

[B26] Lio P. A. Kaye E. T. (2004). Topical antibacterial agents. *Infect. Dis. Clin. North Am.* 18 717–733. 10.1016/j.idc.2004.04.008 15308283

[B27] Lipsky B. A. Hoey C. (2009). Topical antimicrobial therapy for treating chronic wounds. *Clin. Infect. Dis.* 49 1541–1549. 10.1086/644732 19842981

[B28] Liu Y. Zhou Q. Wang Y. Liu Z. Dong M. Wang Y. (2014). Negative pressure wound therapy decreases mortality in a murine model of burn-wound sepsis involving *Pseudomonas aeruginosa* infection. *PLoS One* 9:e90494. 10.1371/journal.pone.0090494 24587379 PMC3938770

[B29] Lowy F. D. (1998). *Staphylococcus aureus* infections. *N. Engl. J. Med.* 339 520–532. 10.1056/NEJM199808203390806 9709046

[B30] Lu C. W. Lin T. Y. Shieh J. S. Wang M. J. Chiu K. M. (2014). Antimicrobial effect of continuous lidocaine infusion in a *Staphylococcus aureus*-induced wound infection in a mouse model. *Ann. Plast. Surg.* 73 598–601. 10.1097/SAP.0b013e318276d8e7 25310128

[B31] Miller L. G. Eisenberg D. F. Liu H. Chang C. L. Wang Y. Luthra R. (2015). Incidence of skin and soft tissue infections in ambulatory and inpatient settings, 2005-2010. *BMC Infect. Dis.* 15:362. 10.1186/s12879-015-1071-1070 26293161 PMC4546168

[B32] Plaut R. D. Mocca C. P. Prabhakara R. Merkel T. J. Stibitz S. (2013). Stably luminescent *Staphylococcus aureus* clinical strains for use in bioluminescent imaging. *PLoS One* 8:e59232. 10.1371/journal.pone.0059232 23555002 PMC3595258

[B33] Ramakrishnan K. Salinas R. C. Agudelo Higuita N. I. (2015). Skin and soft tissue infections. *Am. Fam. Physician* 92 474–483. 10.1016/j.mpmed.2017.08.008 26371732

[B34] Reichman D. E. Greenberg J. A. (2009). Reducing surgical site infections: a review. *Rev. Obstet. Gynecol.* 2 212–221. 10.3909/riog0084 20111657 PMC2812878

[B35] Schierle C. F. De La Garza M. Mustoe T. A. Galiano R. D. (2009). Staphylococcal biofilms impair wound healing by delaying reepithelialization in a murine cutaneous wound model. *Wound Repair. Regen.* 17 354–359. 10.1111/j.1524-475X.2009.00489.x 19660043

[B36] Shaw A. G. Vento T. J. Mende K. Kreft R. E. Ehrlich G. D. Wenke J. C. (2013). Detection of methicillin-resistant and methicillin-susceptible *Staphylococcus aureus* colonization of healthy military personnel by traditional culture, PCR, and mass spectrometry. *Scand. J. Infect. Dis.* 45 752–759. 10.3109/00365548.2013.816439 23957540

[B37] Stevens D. L. Bisno A. L. Chambers H. F. Dellinger E. P. Goldstein E. J. C. Gorbach S. L. (2014). Executive summary: practice guidelines for the diagnosis and management of skin and soft tissue infections: 2014 update by the infectious diseases society of America. *Clin. Infect. Dis.* 59 147–159. 10.1093/cid/ciu444 24947530

[B38] Suaya J. A. Mera R. M. Cassidy A. O’Hara P. Amrine-Madsen H. Burstin S. (2014). Incidence and cost of hospitalizations associated with *Staphylococcus aureus* skin and soft tissue infections in the United States from 2001 through 2009. *BMC Infect. Dis.* 14:296. 10.1186/1471-2334-14-296 24889406 PMC4060579

[B39] Tatiya-aphiradee N. Chatuphonprasert W. Jarukamjorn K. (2016). In vivo antibacterial activity of *Garcinia mangostana* pericarp extract against methicillin-resistant *Staphylococcus aureus* in a mouse superficial skin infection model. *Pharm. Biol.* 54 2606–2615. 10.3109/13880209.2016.1172321 27180784

[B40] Thompson M. G. Black C. C. Pavlicek R. L. Honnold C. L. Wise M. C. Alamneh Y. A. (2014). Validation of a novel murine wound model of *Acinetobacter baumannii* infection. *Antimicrob. Agents Chemother.* 58 1332–1342. 10.1128/AAC.01944-1913 24342634 PMC3957858

[B41] Tong S. Y. C. Davis J. S. Eichenberger E. Holland T. L. Fowler V. G. (2015). *Staphylococcus aureus* infections: epidemiology, pathophysiology, clinical manifestations, and management. *Clin. Microbiol. Rev.* 28 603–661. 10.1128/CMR.00134-114 26016486 PMC4451395

[B42] Turner K. H. Everett J. Trivedi U. Rumbaugh K. P. Whiteley M. (2014). Requirements for *Pseudomonas aeruginosa* acute burn and chronic surgical wound infection. *PLoS Genet.* 10:e1004518. 10.1371/journal.pgen.1004518 25057820 PMC4109851

[B43] Turner N. A. Sharma-Kuinkel B. K. Maskarinec S. A. Eichenberger E. M. Shah P. P. Carugati M. (2019). Methicillin-resistant *Staphylococcus aureus*: an overview of basic and clinical research. *Nat. Rev. Microbiol.* 17 203–218. 10.1038/s41579-018-0147-144 30737488 PMC6939889

[B44] Vermillion M. S. Ursin R. L. Kuok D. I. T. Vom Steeg L. G. Wohlgemuth N. Hall O. J. (2018). Production of amphiregulin and recovery from influenza is greater in males than females. *Biol. Sex Differ.* 9 1–12. 10.1186/s13293-018-0184-188 30012205 PMC6048771

[B45] Wang X. Ge J. Tredget E. E. Wu Y. (2013). The mouse excisional wound splinting model, including applications for stem cell transplantation. *Nat. Protoc.* 8 302–309. 10.1038/nprot.2013.002 23329003

[B46] Zhao G. Hochwalt P. C. Usui M. L. Underwood R. A. Singh P. K. James G. A. (2010). Delayed wound healing in diabetic (db/db) mice with *Pseudomonas aeruginosa* biofilm challenge: A model for the study of chronic wounds. *Wound Repair. Regen.* 18 467–477. 10.1111/j.1524-475X.2010.00608.x 20731798 PMC2939909

