# Supplementary figures and images for: In situ Treatment With Novel Microbiocide Inhibits Methicillin Resistant Staphylococcus aureus in a Murine Wound Infection Model
Source: Front Microbiol. 2020 Jan 23;10:3106. doi: 10.3389/fmicb.2019.03106 (PMC6990143; doi:10.3389/fmicb.2019.03106)

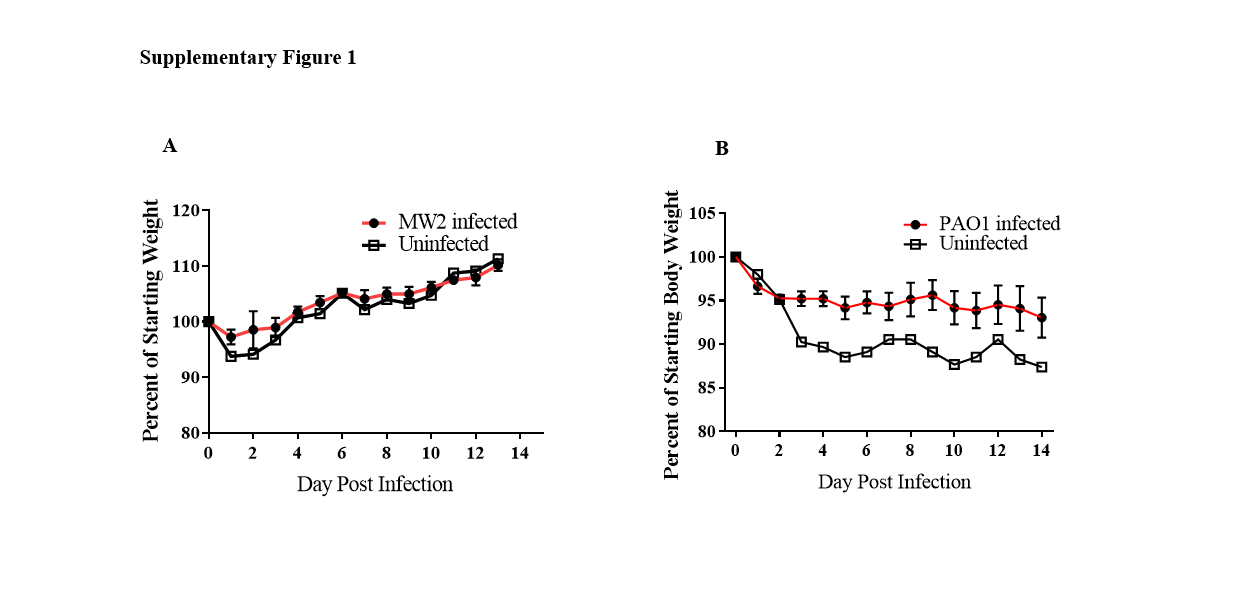

Supplement: FIGURE S1 — Wound infection with CA-MRSA and Pseudomonas aeruginosa does not affect weight change. Mean weights of mice infected with 105 CFU of either CA-MRSA (N = 3) (A) or P. aeruginosa (N = 4) (B). [file Image_1.TIFF]
